# Interactive virtual reality training to improve socio-emotional functioning in adolescents with developmental language disorders: A feasibility study

**DOI:** 10.1177/13591045231220694

**Published:** 2023-12-21

**Authors:** Elke Arts, Bram O De Castro, Ellen Luteijn, Ben Elsendoorn, Constance TWM Vissers

**Affiliations:** 1 Behavioural Science Institute, 6029 Radboud University, The Netherlands; 2 Kentalis Academy 100511 , Royal Kentalis, The Netherlands; 3 Research institute of Child Development and Education, 1234 University of Amsterdam, The Netherlands; 4 Secondary School for Special Education for Children and Adolescents with Language and Communication Problems, 100511 Royal Kentalis, The Netherlands

**Keywords:** Virtual reality, socio-emotional functioning, language, social communication, developmental language disorders, training

## Abstract

Methods to effectively improve socio-emotional functioning by adolescents with developmental language disorders (DLD) are scarce. Current methods to improve socio-emotional functioning in adolescents with other neurobiological disorders seem less suitable, as these methods are highly language based. This study therefore examined the feasibility of the virtual reality (VR) training for socio-emotional skills: ‘InterAction’. The aims of the present study were to (1) examine whether interactive VR is a feasible training method for adolescents with DLD; (2) investigate adolescents’ appreciation of the VR training; (3) examine whether the virtual reality training facilitates the participants’ sense of presence during social practice situations in an interactive digital world; and (4) explore whether adolescents socio-emotional skills improved during the six-session training. A sample of nine adolescents (13–16 years) with DLD reported on their presence in VR contexts and their appreciation toward the VR training. They also completed weekly self-reports on their socio-emotional functioning. Results indicated that ‘InterAction’ was a feasible method to practice socio-emotional functioning with adolescents with DLD. Adolescents highly appreciated the VR training. In addition, adolescents rated the sense of presence as high in the VR training. The individual trajectories showed that improvements in the trained skills varied both between and within participants. The results were also not uniform between the specific skills trained. The findings suggest that interactive virtual reality training may be a promising tool for improving socio-emotional functioning in adolescents with DLD. Future studies should examine the positive indications of this study in a larger sample.

## Introduction

Adolescents with developmental language disorder (hereafter: DLD) are at greater risk of socio-emotional problems than typically developing (TD) peers. Adolescents with DLD often encounter socio-emotional challenges, such as increased levels of shyness and anxiety in social settings, fewer positive peer relations, and greater risk of victimization, social isolation and depression (Conti-ramsden et al., 2013; Durkin et al., 2017; Smit et al., 2019). These problems in adolescents with DLD indicate the need to improve their socio-emotional functioning. However, methods effectively training socio-emotional functioning by adolescents with DLD are scarce (Arts et al., 2022). Moreover, current methods to improve socio-emotional functioning in adolescents with other neurobiological disorders, often ask to reflect on hypothetical vignettes (Bauminger, 2002; Laugeson et al., 2012). This methods seem less suitable for youth with DLD, as these hypothetical vignettes are highly language based. This study therefore examined the feasibility of a more behaviourally focused interactive virtual reality training (i.e. InterAction). Adolescents with DLD encounter difficulties in their receptive and/or expressive language skills, with no discernible medical origin (Bishop et al., 2017). Expressive language involves the ability to vocalize and convey clear communication through speech, whereas receptive language involves the skill of comprehending and understanding spoken language (Arts et al., 2022). It is estimated that DLD has a prevalence of 5–10% (Law et al., 2000; Van Agt et al., 2011). DLD is more common in boys than girls (ratio 3:1) (Wiefferink et al., 2020).

Social and emotional functioning are closely intertwined. Emotional competence evolves through the process of emotion socialization, wherein children acquire the ability, via social interactions, to identify, comprehend, manage and convey emotions in accordance with the societal norms of their environments (Saarni, 1999). The social domain encompasses the acquisition of insights and skills that enable positive interactions with others (Vissers et al., 2021). Research indicates that in the social domain, ‘social contacts with peers’ was most frequently reported as problematic among adolescents with DLD, which was related to pragmatic language problems (Mok et al., 2014). Adolescents with DLD did not report other aspects of social functioning (e.g. prosocial behavior) as problematic (Conti-Ramsden et al., 2013).

To develop effective training for socio-emotional functioning of adolescents with DLD, it is necessary to understand the etiology of their problems with social functioning. From a neuropsychological perspective (Tomas & Vissers, 2019), the socio-emotional problems of individuals with DLD can be explained by cognitive difficulties in mentalization (Theory of Mind [ToM], Bakopoulou & Dockrell, 2016; Nilsson & de López, 2016) and self-regulation (Executive Functioning [EF], Henry et al., 2012; Sikora et al., 2019) EF can be described as the set of advanced cognitive control processes, to control human cognition and behaviour, in pursuit of specific goals (Diamond, 2013; Miyake et al., 2000). ToM encompasses the capacity to understand emotions (affective), thoughts, intentions, desires and beliefs (cognitive) of oneself (intrapersonal) and others (interpersonal) (Westby & Robinson, 2014).

There are three suggested models to explain the interplay between the language and cognitive problems. The first model proposes that cognitive factors stimulate the development of language. In this way, ToM and EF are proposed to allow children to learn new words (Vugs et al., 2015). In the second model, language facilitates the development of ToM and EF. Following this approach, EF and ToM are positively influenced by the use of self-regulatory speech and conversations about others’ thoughts, intentions and feelings (Milligan et al., 2007). The third model proposes that language and problems in EF and ToM co-occur as a product of the same underlying factors (Bishop et al., 2014).

Well-developed EF and ToM skills are indispensable to adequately interact in social situations. Individuals are expected to understand and predict others’ behaviour and to represent mental states of oneself and others (i.e. ToM) (Arts et al., 2022; Smit et al., 2019). Furthermore, adequate socio-emotional functioning relies on the presence of well-established self-control functions (Arts et al., 2022; Mcquade et al., 2013). For example, individuals need to inhibit their distractive thoughts and inappropriate behaviour (inhibition), demonstrate flexibility in varying social situations (cognitive flexibility), and cognitive capacity to process social information (working memory).

Once socio-emotional problems arise, they may activate a vicious cycle: Less frequent and efficient social dialogue can result in less sufficient social input and practice with the underlying aspects of interaction and communication, that may in turn further compromise social interactions adolescents could have learned from (Vissers et al., 2021). Indeed, by 16 years of age, nearly 40% of individuals with a history of DLD appear impaired in their interactions with peers (St Clair et al., 2011). Adolescents with DLD are more likely to experience bullying victimization and exhibit more difficulties in peer relations and friendships (Conti-Ramsden et al., 2013; Durkin & Conti-ramsden, 2010). In addition, they are less socially confident, experience higher levels of shyness, lower levels of social self-efficacy (Durkin et al., 2017), and show more withdrawal in social situations (Hart et al., 2004), again resulting in fewer learning opportunities within social interactions and socio-emotional problems.

This vicious cycle of deficits underscores the need for an intervention method to improve the underlying components of socio-emotional functioning in adolescents with DLD, and which provides opportunities to practice social interactions with peers.

### Practicing socio-emotional functioning

While most intervention methods for individuals with DLD focus on expressive or receptive language abilities, improvement of social skills and the ability to socially interact with peers has received increased attention (Law et al., 2017). Unfortunately, very little data exists on effects of training socio-emotional functioning in adolescents with DLD (Arts et al., 2022). Despite these limited data, several suggestions for improving socio-emotional functioning in individuals with DLD have been made in the literature. First, methods should focus on the linguistic, cognitive, and social communication skills that underlie socio-emotional functioning (Vissers et al., 2015; Vissers & Koolen, 2016). For example, the ability to understand what others say and to formulate an appropriate response (linguistic skills), are necessary to participate in social interaction. Simultaneously, understanding the intentions and emotions of peers (ToM) seems equally important to adequately interact with each other (Bishop et al., 2000).

Second, it is suggested that individuals, to achieve adequate social communication skills, need to learn from many social interactions with different peers, in different situations and with different pragmatic rules (Vissers et al., 2021). Important skills of social interaction include: turn taking, repair of communication breakdowns, responding to questions, topic management skills (Gerber et al., 2012), entering peer groups, and negotiating and resolving conflicts (Timler et al., 2005). To achieve a generalization effect, intervention methods should ensure that these social communication skills are trained in meaningful, natural contexts with peers (Timler et al., 2007).

To achieve adequate cognitive skills it seems also crucial to participate in social dialog (Carpendale & Lewis, 2004; Kuhn et al., 2014). Within this social dialog, adolescents will learn about different perspectives and beliefs of others. This process will provide the basis for the capacity to further adopt and internalize perspectives of others, and thus the development of ToM (Fernyhough, 2008). Studies focusing on improving ToM in children suggest to specifically train the meaning of emotion words (Avila, 2019), to practice mental state verbs (Durrleman, 2020) and analyze video-clips of social interactions to help adolescents recognize conversational cues and perspectives in real people (Westby & Robinson, 2014). For EF, it has recently been suggested that lab-based executive-function training may not be effective, at least not to obtain far transfer to daily social life (Kassai et al., 2019). Based on these recent insights, it has been suggested to train EF in real-life, meaningful, social contexts (Diamond & Lee, 2011).

### The potential benefits of VR

Since many individuals with DLD experience bullying victimization and exhibit more difficulties in peer relations and friendships (Conti-Ramsden et al., 2013; Durkin & Conti-ramsden, 2010), practice and support for social behaviour in real-life practice seems difficult to attain within practical and ethical boundaries. Fortunately, the utilization of virtual reality (VR) has been proposed as a possible way to address these problems by emerging participants in realistic social learning experiences. Recent studies showed favorable outcomes (participants and clinicians highly appreciated the VR setting; only reported minor technical issues) of using interactive VR for improving socio-emotional functioning in youth with autism (Didehbani et al., 2016; Kandalaft et al., 2013), and aggressive behaviors (Alsem et al., 2021). Based on these results, virtual reality may also be a promising technique for adolescents with DLD (Arts et al., 2022).

The use of virtual reality training has several advantages. The main advantage is that VR can offer immersive, interactive and dynamic real-life scenarios and enable social interactions remarkably similar to the real world (Veling et al., 2014). This real-world experience may increase the generalization of the practiced skills. Another attraction is that the real-world environment in VR eliminates the need for complex linguistic and mentalizing skills involved in the dialogues about hypothetical social interactions that are common in traditional treatment, so that adolescents with DLD may have more cognitive resources left at their disposal for social reflection and task attention (Brinton & Fujiki, 2006). Moreover, VR provides a safe and reliable environment: adolescents can practice without any chance of victimization, rejection, embarrassment or other risks that are associated with real-life situations (Didehbani et al., 2016). Therefore, it seems well-suited for adolescents with DLD, who have experienced negative social interactions. At the same time, VR is controllable, whereby social situations can be paused, repeated and adjusted to personalized preferences or level (Nijman et al., 2019). In the end, utilizing virtual reality for therapeutic treatment in adolescents may boost their motivation, as most adolescents are intrigued by computers, video games, and innovative technologies (Brezinka, 2014; Didehbani et al., 2016).

In contrast with the proposed benefits, it is also possible that some adolescents may not fascinated by computers and video games. Furthermore, research has historically delved into the negative facets of VR, such as cybersickness (i.e. headache, nausea, dizziness) and technological difficulties (e.g. computer errors). However, these negative symptoms were mainly prevalent during an era of slower hardware and older technology. Recent research showed that clinicians and individuals in recent studies no longer reported these adverse facets of VR (Alsem et al., 2021; Nijman et al., 2020; Verhoef et al., 2021). Nevertheless, it is possible that some individuals do continue to see certain disadvantages in using VR training.

### Characteristics of virtual reality

VR is characterized by the experience of a sense of presence (i.e. the sense of being there) in an interactive digital world. The computer generates an image, which is presented to the user by means of VR glasses in a three-dimensional way (Veling et al., 2014). The movements of the user are registered by a tracker and translated into the displayed image in the VR glasses. The outcome is an immersive experience that evokes psychological an physiological responses remarkably to those in the real world (i.e. sense of presence) (Veling et al., 2014). When the possibilities of VR are used to create role-playing and naturalistic situations, it is hypothesized that VR could support generalization of trained skills to real-life contexts (Parsons & Mitchell, 2002).

The sense of presence has usually been considered as the key element of VR (Baños et al., 2004). To achieve this sense of presence, recent studies suggest to make use of immersion and affective content (Baños et al., 2004; Gorini et al., 2011). Immersion refers to the objective description of the used technology (i.e. system resolution, software, speed of images, sound). Affective content refers to the emotional conditions (i.e. narratives, emotions/voice/sayings of the characters) that are added to the virtual environments. Thus, to achieve some feelings of presence in individuals using VR, the training must consist of a combination of adequate technology and affective content.

### The present study

Given the feasibility (in other clinical populations) and potential advantages of using virtual reality, we decided to develop an individual virtual reality training to practice socio-emotional functioning. To the best of our knowledge, this is the first study examining the feasibility of a virtual reality training in adolescents with DLD. Hence, we decided to conduct a small-scale feasibility study. The aims of the present study were to (1) examine whether this interactive VR is a feasible training method for adolescents with DLD; (2) investigate adolescents’ appreciation of the VR training; (3) examine whether the virtual reality training facilitates the participants sense of presence in social practice situations; and (4) explore whether adolescents socio-emotional skills increased during the six-session training.

## Method

### Participants

Nine adolescents with DLD (*3 girls, 6 boys*), were recruited at two secondary schools for special education of children with communication problems in the Netherlands. These schools are specialized in educating children who are deaf or hard of hearing and children with DLD. All participants met the inclusion criteria for InterAction: age 12–18 years (*M = 14.4)*, an established developmental language disorder and no history of epilepsy. To recruit these participants, all students with DLD (whose casefiles met the studies inclusion criteria and exhibited issues with socio-emotional functioning [<3.0 in at least one category of the IKAN] see Appendix B, Table B1) received an invitation letter to participate in the study. This allowed selected adolescents to voluntarily enroll in the research (i.e. voluntary sampling). Adolescents were recruited until the predetermined sample size was reached. Written informed consent was obtained from 9, out of a total of 15 approached participants and their parents/caregivers (60%). Participation was voluntary and adolescents and parents were assured of confidential use of their data.

### Diagnosis

All adolescents were indicated as having DLD by a multidisciplinary team at an audiological center. To obtain a diagnosis of DLD, adolescents had to have (1) no severe hearing problems, as assessed by an audiologist; (2) typical nonverbal intelligence, as assessed by a psychologist; (3) no neurological problems; and (4) severe and persistent language difficulties, impacting communicative efficacy (SIAC, 2023). To determine the severity and content (domains: speech, grammar, semantics, pragmatics) of language problems, a speech-language therapist at the audiological center used the Clinical Evaluation of Language Fundamentals (CELF-5; Wiig et al., 2019), The Schlichting Test for Language Comprehension (Schlichting et al., 2010a), the Schilting Test for Language Production (Schlichting et al., 2010b) and the Peabody Picture Vocabulary Test (PPVT; Schlichting, 2005). Language problems were only ‘severe’ enough for a DLD diagnosis, when individuals (1) score ≤2 SD below average in one of the four language domains, or (2) score ≤1.5 SD below average in two of the four language domains, or (3) score ≤ −1 SD below average in three of the four language domains. This deviation must be demonstrated with at least two language tests.

### Procedure

Each participant completed one session, 50 minutes, once a week, for six weeks. Every week, approximately 5 minutes were used to fill in the weekly questionnaire of socio-emotional functioning (see below), 5 minutes to rate how much they appreciated the training and how immersive the training was, and 40 minutes for the InterAction training itself. The InterAction training was performed in a small classroom, at their own schools, during class time, with a table for the complete VR system, where the participant could walk around freely while wearing the VR glasses.

### InterAction training

InterAction is a newly developed training method to improve socio-emotional functioning with interactive virtual reality. InterAction consists of six weekly, individual sessions of approximately 40 minutes. All sessions were conducted by the same experimenter. The experimenter has an background in education and behavioral sciences. The experimenter underwent a comprehensive full-day training on the VR technology used in InterAction. The training was facilitated by CleVR (the software company of InterAction).

The sessions of InterAction were very structured (see Appendix A, Table A1). First, each session started with mentioning the target skill (see Table 1: content per session). Second, the experimenter activated the adolescent’s prior knowledge by asking some questions (e.g. which conversation topics do you already know?). Third, the adolescent watched role-playings of other peers performing the session skill (i.e. videoclips on a laptop). The videoclips consisted of three social interactions (two wrong, and one good example of the performed/trained skill). Watching these videoclips allowed the experimenter to pause the clip, rewind, and replay, offering the possibility to focus on perspectives of both characters (Westby & Robinson, 2014). Fourth, the adolescent answered the following questions: (1) “What did the characters do well in the video?”, (2) “Which skills need to be improved in the characters?”, (3) “What would both characters think?”, and (4) “What would both characters feel?”. Fifth, adolescents practiced the skill verbally with the therapist (e.g. “Which questions can you ask peers about their vacation?”). Sixth, the adolescents practiced the learned skill twice in the VR environment. Finally, the adolescents reflected on their practiced skills in the VR.

**Table 1.** Content of the interaction training sessions.

Skill per session	Content*	Virtual environment**
1. Taking initiative	Adolescent learns how to start a conversation, and how to introduce themselves to peers.	Schoolyard, park
2. Asking questions	Adolescent learns how to ask questions, and talk about the same interests with peers.	Workspace, backyard
3. Having a conversation	Adolescent learns several conversation topics and how to ask different (follow-up) questions about these topics.	Bus, living room
4. Knowing your own feelings	Adolescent learns how to tell peers when he/she is nervous, disappointed or angry.	Park, bedroom
5. Recognize feelings of another	Adolescent learns how to react adequately to peers who are nervous, disappointed and angry.	Workspace, schoolyard
6. Standing up for yourself	Adolescent learns how to react to harassing peers, and how to keep calm when peers are angry.	Schoolyard, backyard

*For additional visual material about the VR training content (InterAction) click this link [Dutch version only]: https://www.youtube.com/watch?v=esc16t-fObU

** For additional visual material about the virtual environment (made by CleVR) click this link: https://www.youtube.com/watch?v=nOUx4UuEDT0

### Interactive virtual reality

**Figure 1.**
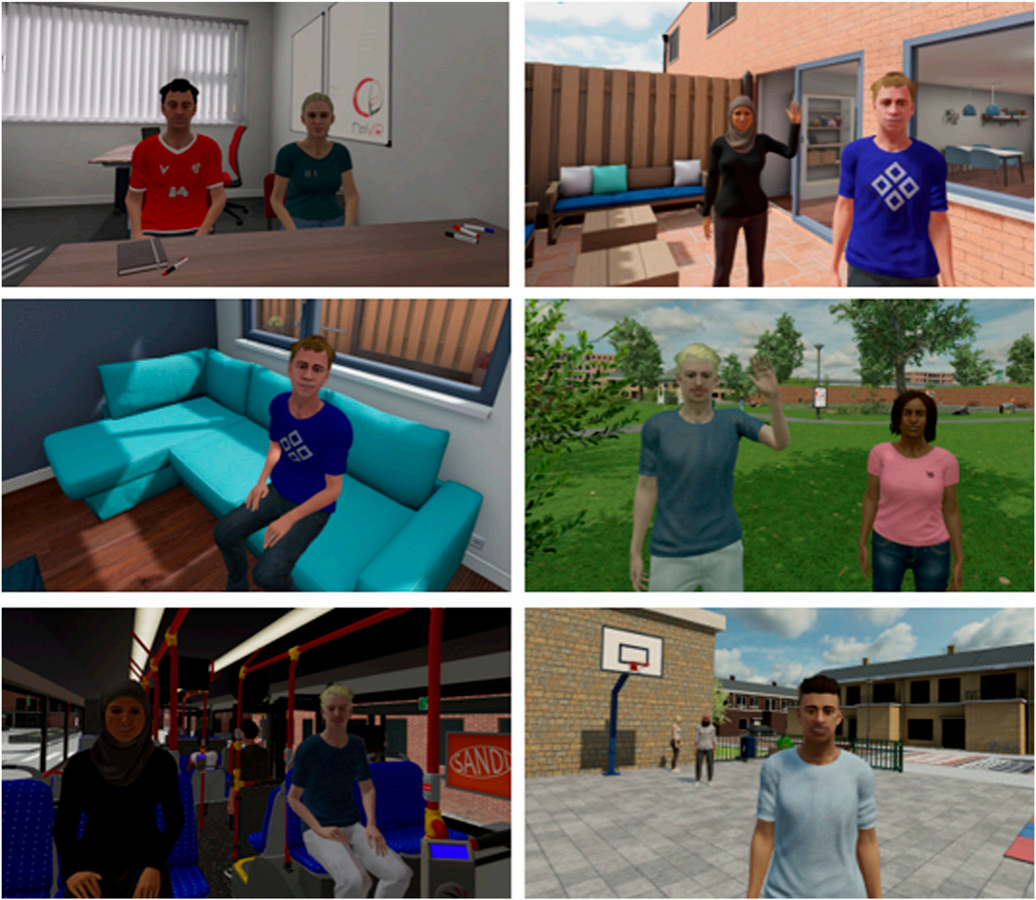
Virtual reality workspace, backyard, living room, park, bus, and schoolyard.

The content of the VR scenarios and interactive peer interaction was based on several conversations with teachers, speech therapists, psychologist and adolescents with DLD. The InterAction VR environment consisted of a workspace, a schoolyard, a park, a bus and a house (see Figure 1). Within these various environments, adolescents had the opportunity to communicate with digital peers. The VR software was developed by CleVR BV (https://clevr.net). Participants wore an Oculus Rift S (designed by Facebook Technologies and Lenovo), with a resolution of 1280 × 1440 per-eye, with an approximate diagonal field of view of 115. To hear sounds and voices in the VR, participants wore a headphone (3M Peltor WorkTunes Pro). Head tracking was realized by the built-in-sensors (6DOF inside-out tracking through 5 built-in cameras) of the Head Mounted Display (HMD).

The VR training world included eight virtual peers, that differed in their haircut, skin color, faces, height, and clothes. All virtual peers were provided with a name and a short narrative about their identity/life (i.e. family, hobby’s). The verbal responses and movements of virtual peers were controlled by the experimenter. We did not script the social interactions, to provide the spontaneity of natural communication (Didehbani et al., 2016). The voice of the experimenter was, via the microphone (using the program MorphVox), transformed into teenage voices of the characters, so the experimenter could spontaneously adapt all characters’ behaviors to the participant’s behavior. The emotions of the characters, during the social interactions, were controlled by the experimenter. Present emotions in the VR were: sad, angry, happy, scared and disgust. By adding names, teenage voices, emotions and narratives about the characters identity/life, we tried to add affective content to the VR training, and thus maximize the sense of presence in participants. During the sessions, adolescents practiced the session’s skill in different digital environments, with different digital characters and different peer emotions. By varying these parameters adolescents may develop flexibility in their thinking (i.e. understand why a social strategy that might work in one situation may not work in another), thus promoting far transfer (Westby & Robinson, 2014).

### Measures

The feasibility of InterAction was assessed by investigating several topics, such as: training implementation and suitability, appreciation of the training and preliminary participant results (Orsmond & Cohn, 2015). Specifically for the efficacy of the VR, we also monitored participants sense of presence within the virtual environment.

#### Training implementation and suitability

The first topic, training implementation, included the usability of the used technology and the occurrence of any technical issues (e.g. tracking problems, difficulties with headset/microphone, computer errors or other unforeseen problems) regarding the VR system. In addition this topic included, suitability, related to training duration, practice time in VR, and the fact if adolescents experienced any negative effects of VR (e.g. cybersickness). To investigate this topic, the experimenter was asked qualitative questions about his/her experience after each training session (e.g. “*Have there been any unexpected technical issues? If so, which ones? Can the training sessions be conducted in an ethical manner (e.g. cybersickness) If not, what ethical issues occurred?”*).

#### Appreciation of the training

To measure the second topic, appreciation of the training, the adolescents completed a weekly (self-designed) assessment. Adolescents assessed (paper-and-pencil) their appreciation of the training on two items (i.e. “*I liked today’s training”* and *“I’m looking forward to the next training”*) on a 3-point Likert scale (“*not – a little bit – yes”*). Ratings were averaged over both items.

#### Sense of presence

Adolescents rated (paper-and-pencil) their sense of presence on three items (i.e. *“the VR world is realistic”, “the contact with the VR peers feels realistic”, “During the VR, the exercise had my full attention”)* on a 3-point Likert scale (“*not – a little bit – yes”*)*.* Ratings were averaged for all three items.

#### Preliminary participant results

Adolescents assessed their socio-emotional functioning for six weeks, before each training session. To assess weekly change in adolescents socio-emotional functioning, a questionnaire was needed that could be used for weekly measurements. Therefore, we choose to use a new custom-made digital questionnaire. This custom-made questionnaire consists of eight items (see Table 2). Adolescents specified their level per item of socio-emotional functioning by indicating a position along a continuous line between two end points (i.e. Visual Analogue Scale). The two end points consisted of “*I can do it well”* and “*I can’t do it”*.

**Table 2.** Content of custom-made questionnaire.

Abbreviation	Asked item
1. Conversation	I Can have a conversation with peers I barely know
2. Question	When someone asks me something, I can ask a question back
3. Going to do	I Can tell peers what I’m going to do
4. Been through	I Can tell peers what I have been through
5. My feelings	I Can share my feelings with peers
6. Feelings of others	I Can support peers with their feelings
7. Want	I Can tell peers what I want
8. Don’t want	I Can tell peers what I don’t want

#### Analyses

To describe feasibility, training appreciation, and sense of presence, we used descriptive statistics. To explore training effects on socio-emotional functioning, we evaluated plots of within-person change over time. As our small-sample study was not designed to examine statistical significance of changes over time at the group level, no statistical tests were conducted.

## Results

### Training implementation and suitability

Within the area of training implementation, the experimenter reported minor technical issues regarding the VR technology (i.e. three sound- and tracking issues; were resolved after restarting the system). These issues could (according to the experimenter) be prevented by starting the VR system before the participants are present; this increases the implementation of the training. In addition, findings reveal that the VR training was suitable for adolescents with DLD in school setting. The experimenter did not report any negative effects of the VR on the participants. Moreover, no uncertainties were reported regarding the assignments in the InterAction workbook. The assignments in the workbook clarified the expectations for the adolescents in the virtual environment (experimenter’s report). During the 40 minutes training sessions, participants practiced an average of 8 minutes and 57 seconds per session in the VR environment with a total average of 52 minutes and 42 seconds per participant over the 6 weeks of training. Finally, the experimenter expressed that a more extended training duration (50 minutes per session) results in more practice time in VR (i.e. the experimenter had to frequently end the virtual conversation due to the training time reached).

### Appreciation of the training

Adolescents appreciation of the VR training was very high. The scores of the adolescents are presented in Table 3.

**Table 3.** Results on appreciation and sense of presence.

Category	Asked item	Not	A little bit	Yes	Mean	S.D.
Appreciation	I Liked the training	0 (0%)	1 (1.9%)	53 (98.1%)	2.98	.14
	I’m looking forward to the next training	0 (0%)	7 (13%)	47 (87%)	2.87	.34
Sense of presence	The VR world is realistic	0 (0%)	22 (40.7%)	32 (59.3%)	2.59	.50
	The contact with the VR peers feels realistic	0 (0%)	15 (27.8%)	39 (72.2%)	2.72	.45
	I Was completely focused during the VR exercise	0 (0%)	9 (16.7%)	45 (83.3%)	2.83	.38

### Sense of presence

Adolescents rated the sense of presence in the VR environment relatively high. The scores of the adolescents are presented in Table 3.

### Preliminary participant results

**Figure 2.**
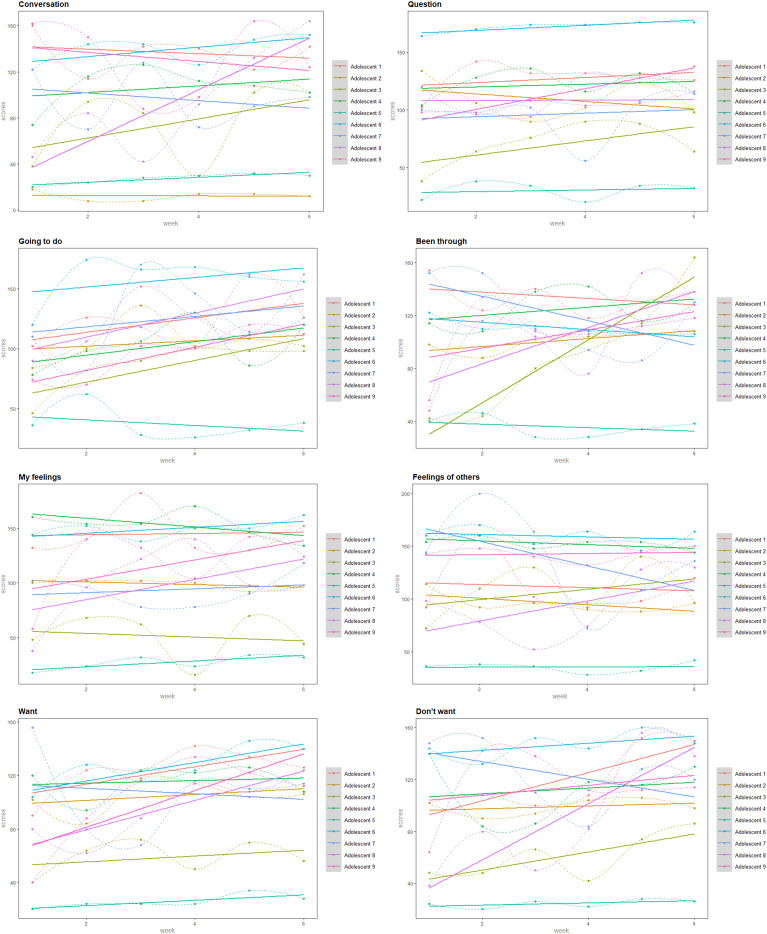
Socio-emotional functioning reported by adolescents over weeks. Dots reflect reported scores, and solid lines reflect linear trendlines.

We created plots to explore within-person change in the eight assessed socio-emotional skills over the six training weeks, based on adolescent reports (see Figure 2). These plots demonstrate that improvements in the trained skills were not uniform between and within participants. Some participants showed clear change – other patterns were less convincing. For example, the trendlines of adolescent 7 showed an increase over time in only three trained skills, compared to the increased trendlines of adolescent 8 on seven socio-emotional skills. In addition to between-person differences, the results also show within-person differences. For example, the trendlines of adolescent 6 indicated an increase on six trained socio-emotional skills. In contrast, the linear trendlines of the other two skills suggested a decrease in socio-emotional skills over time. This phenomenon occurred for all participating adolescents.

The results of the study were also not uniform per trained skill. This is also visually illustrated in Figure 2. For example, the linear trendlines of eight adolescents showed improvements on the trained skill ‘*telling what I’m going to do’.* However, the trendline of one participant showed a decrease on this trained skill. This heterogeneity applies to all trained skills.

## Discussion

This study assessed the feasibility of an interactive virtual reality training to improve socio- emotional functioning in adolescents with DLD. The first aim of the study was to examine whether interactive virtual reality is a feasible treatment method for adolescents with DLD. Results of this study showed that VR seems a feasible method since (1) adolescents with DLD reported no negative effects over six weeks of training, and (2) there were no technical issues noted during preparation and implementation of the VR sessions. The second aim was to investigate adolescents’ appreciation of the VR training. Results showed that adolescents’ training appreciation was very high, with maximum scores for all sessions by almost all participants. The third aim was to examine whether the virtual reality training facilitates the participants’ sense of presence in social situations. Results indicate that adolescents rated the aspects of the VR as realistic, and that they felt themselves mentally involved in practice moments, contributing to a high sense of presence. The fourth aim was to explore whether adolescents’ socio-emotional functioning improved during the six-session training. Weekly training progress based on self-reports showed that improvements in the trained skills did occur, but were not uniform between and within participant, nor per trained skill.

We assume that the variability in change on the trained skills reflects the complex nature of factors underlying socio-emotional functioning, and associated developmental profiles in adolescents with DLD. DLD is a heterogeneous category that encompasses a wide range of problems and may be associated with very different social problems in different clients (Bishop et al., 2017). The difference in the problems experienced during social situations may contribute to the varying results. Given the diagnostic heterogeneity and complexity of DLD, it seems an important step, for future studies, to focus on individuals in their unique contexts (e.g. measure improvements of individuals, tailored training, personal treatment goals). This can be achieved, for example, by having the adolescents indicate up to 5 problems in socio-emotional functioning that they are concerned about (Chiu et al., 2022). Based on these individually formulated problems, weekly measurements can be used to determine whether the adolescents experience progress on these goals.

In addition, it was notable that the scores within adolescents did not improve or decrease consistently, but fluctuated over the training period. We tentatively suggest that this fluctuation of scores may have been influenced by the experience of positive and negative events in adolescents’ daily lives (e.g. illness). To avoid excessive influence of such fluctuations on evaluations, future studies could add more measurement occasions in both the baseline and intervention period. By adding these datapoints, one specific event will have less influence on the whole pattern of change.

The weekly indicators of social functioning were self-reports of socio-emotional functioning by adolescents with DLD. Since these self-reports were the only measurement technique in this study, interpreting the results warrant caution. It has been suggested that self-appraisal is dependent upon frontal lobe development, which may continue to mature into early adulthood (Hughes et al., 2009). Hence, it is possible that the self-report measure of socio-emotional functioning was not as representative of actual behaviour as observational measures would have been. Indeed, a recent study in adolescents with DLD showed that adolescents rated themselves more positively than did their parents (Hughes et al., 2009). In conclusion, weekly self-reports may not be as valid for measuring social-emotional functioning. Future studies are recommended to add measurements from multiple perspectives (parents/teachers), to gain a more accurate reflection of socio-emotional functioning.

### Limitations and recommendations

It is worth re-emphasizing at this point that this is not an efficacy study. Limitations of design (i.e. absence of baseline period or control group) and small sample size limit the generalization of findings. Future studies aiming to evaluate the effects of VR-based training for socio-emotional functioning should examine its impact with a larger sample of adolescents with DLD. Second, because the study lacked a control condition, it is uncertain whether changes in socio-emotional functioning were an actual effect of the training. To determine this, future studies could use control groups or use single-case data that include a control phase (i.e. baseline period) to examine training effects. Moreover, more datapoints obtained during both baseline and intervention periods would minimize the influences of incidental life events on the pattern self-reported functioning over time A third limitation is that the current study utilized self-reports of adolescents with DLD as primary outcome measures. Additional measurements from another perspective (i.e. parents/teachers) are recommended in future studies. Additionally, we did not use a standardized measures to assess presence and socio-emotional functioning, because we were interested in elements specific to our intervention. Furthermore, the items in these questionnaires are not formulated to capture short term changes (as they typically ask for the past year or months). Thus, while our custom surveys were informative for further development of the VR training, it cannot be directly compared with previous research. To evaluate the training outcomes, we recommend future studies to use psychometrically established assessment measures. Finally, the relatively short training period may influenced the outcome of the VR training on socio-emotional functioning. Studies indicate that in the context of cognitive training, increased treatment intensity could lead to more favorable outcomes (Choi & Medalia, 2005). Future studies may extend the training time per participant.

### Conclusions

In conclusion, the results suggest that interactive virtual reality is a feasible method for training socio-emotional functioning, that is highly appreciated by participating youth. In addition, VR training meets the need for an interactive training method for youth with DLD. However, as an uncontrolled feasibility study, the results of this study lack statistical power and methodological rigor to draw conclusions concerning the efficacy of InterAction. Given the feasibility and high appreciation of the VR training, a next logical step would be to examine the positive indications of this study in a larger sample, with more tailoring of training to individual needs, a longer period of training, and the addition of a baseline control phase to evaluate effectiveness.

## Supplemental Material

Supplemental Material - Interactive virtual reality training to improve socio-emotional functioning in adolescents with developmental language disorders: A feasibility study Supplemental Material for Interactive virtual reality training to improve socio-emotional functioning in adolescents with developmental language disorders: A feasibility study by Elke Arts, Bram O De Castro, Ellen Luteijn, Ben Elsendoorn and Constance TWM Vissers in Clinical Child Psychology and Psychiatry

## Appendix A : Example of the training session (InterAction)

**Table A1.** Step-by-step explanation of session 3 ‘Having a conversation’.

Training step	Content
1. Introduction to the target skill	The session begins with an introduction to the target skill. Tell the student that today we’re going to work on ‘having a conversation’. Say the following to the student: “There are two specific goals linked to today’s session: 1. You will learn at least three topics to bring up during a conversation with peers; 2. You will learn at least 4 questions to ask about these topics;
2. Activating (prior) knowledge	We continue the session by activating some (prior) knowledge. Say the following to the student: “When you want to have a conversation with someone, it’s helpful to know what you want to talk about. There are certain topics that almost all peers can talk about, for example: Vacation. Think of more topics that you can talk about with peers. Write these topics down in your workbook.” (*possible answers: school, sports, hobbies, weekend plans, vacation*)
3a. Watching role-playings of other peers (videoclip)	Go to: https://elkearts.wixsite.com/interaction [Dutch version only]. Watch the videoclip ‘gesprek voeren’ [having a conversation].
3b. Answering questions about the videoclip	After watching every videoclip (3 in total), you ask the student the following questions about the videoclip: - What did the characters do well in the video? - Which skills need to be improved in the characters? - What would both characters think? - What would both characters feel?
4. Practicing the skill verbally with the therapist	We continue the session with some verbal practice. Tell the student that we are now going to practice which topics we can bring up in a conversation with peers. Say the following to the student: “Think of three topics you can start a conversation about, and write them down in your workbook (*possible answers: school, sports, hobbies, weekend plans, vacation*). After the student has written these topics, say: “For each topic, think of at least four questions that you can ask. Write these questions below the topics”. *For example (conversation topic: vacation):* - *Are you going on summer vacation?* - *Where are you going?* - *Who are you going on summer vacation with?* - *Are you staying in a hotel, or on a campsite?*
Two practice moments in the VR environment	Tell the students that it is now time to practice in the virtual environment. The goal for the student is to engage in a conversation with a virtual peer, and introducing at least three conversation topics. Additionally, the task for the student is to ask the peer at least four questions about these topics. The first practice session is with a boy named ‘Tim’ on a bus. The second practice session is with a girl names ‘Sophie’ in the living room.
Reflection on the practiced skills in VR	After each practice session in VR, it is time for reflection. Ask the student the following questions: - What went well in the VR exercise? - What did not go as well during practice? (*Discuss with the student how this can be done differently or improved*).

## Appendix B: Me-and-the-other-list [IKAN]

To assess the inclusion criteria ‘problems in social communication’ we used the annual school questionnaire [IKAN] (van Hoof et al., 2023), completed by the teachers of the adolescents. This questionnaire consist of 8 categories, with a total of 34 questions. For this study, we used the results of the 4 categories of social communication. Teachers rated each item on a 4-point Likert scale (1 = the student cannot do this yet, 2 = the student can do this with the help of an adult, 3 = the students can do this independently with helpful tools [timer, step-by-step plan], 4 = the student can do this completely independently). Ratings were averaged for all 4 categories. To participate in the VR study, students had to score below the set school norm (<3), in at least one of these categories.

### I. Standing up for yourself

1. The student expresses their opinion and indicates what he/she wants.
2. The student makes their own choices and is willing to share what he/she chooses, even if others choose differently.
3. The student clearly communicates to peers when he/she doesn’t want something or finds it uncomfortable (influence in relation to the peer group).
4. The student asks a question when necessary (a question for help, clarification or information).

### II. Dealing with emotions and behavior

1. The student expresses their feelings to others at the moment they experience them.
2. The student manages their feelings well and makes useful choices.
3. The student reflects on their behavior and explains what they did and how they could have done things differently.
4. The student considers what someone else might think and feel.
5. The students displays behavior that aligns with how someone else is feeling.
6. The student recognizes when they have a conflict or when they don’t understand someone else.
7. The student resolves a conflict/disagreement or misunderstanding.

### III. Aligning with people and the environment

1. The student adjusts their behavior when necessary.
2. The student respects others, even if they have a different culture, skin color, opinion, appearance, sexuality, or gender identity.
3. The student handles changes in a pleasant manner.

### IV. Presenting yourself positively to others

1. The student behaves politely and kindly, and adheres to agreements.
2. The student establishes connections with peers and maintains those connections.
3. The student listens to the other person in a conversation and responds to them.
4. The student manages themselves well on social media and/or the internet.

**Table B1.** Average scores of the participants on the four categories of social communication on the IKAN (filled out by the teachers).

Adolescent	Teacher reported average score
1	3.08
2	2.17
3	3.55
4	3.59
5	3.63
6	3.40
7	2.88
8	2.77
9	2.87

## References

[bibr2-13591045231220694] Alsem S. C. Dijk A. V. Verhulp E. Castro B. O.D. (2021). Using virtual reality to treat aggressive behavior problems in children: A feasibility study. Clinical Child Psychology and Psychiatry, 26(4), 1–14. 10.1177/13591045211026160 PMC8593284 34151602

[bibr4-13591045231220694] Arts E. Orobio de Castro B. Luteijn E. Elsendoorn B. Vissers C. T. W. M. (2022). Improving social emotional functioning in adolescents with developmental language disorders: A mini review and recommendations. Frontiers in Psychiatry, 13, 966008. 10.3389/fpsyt.2022.966008 36569624 PMC9786114

[bibr68-9135910452322] Avila S. A. (2019) *A Social Communication Intervention to Facilitate Emotion Word Learning in School-Age Children with Developmental Language Disorders (2019)* . *All Theses and Dissertations* . 7367. https://scholarsarchive.byu.edu/etd/7367

[bibr6-13591045231220694] Bakopoulou I. Dockrell J. E. (2016). The role of social cognition and prosocial behaviour in relation to the socio-emotional functioning of primary aged children with specific language impairment. Research in Developmental Disabilities, 49–50, 354–370. 10.1016/j.ridd.2015.12.013 26773217

[bibr7-13591045231220694] Baños R. M. Botella C. Alcañiz M. Liaño V. Guerrero B. Rey B. Liaño V. (2004). Immersion and emotion: Their impact on the sense of presence. CyberPsychology and Behavior, 7(6), 734–741. 10.1089/cpb.2004.7.734 15687809

[bibr8-13591045231220694] Bauminger N. (2002). The facilitation of social-emotional understanding and social interaction in high-functioning children with autism: Intervention outcomes. Journal of Autism and Developmental Disorders, 32(4), 283–298. 10.1023/a:1016378718278 12199133

[bibr9-13591045231220694] Bishop D. V. M. Chan J. Adams C. Hartley J. Weir F. (2000). Conversational responsiveness in specific language impairment: Evidence of disproportionate pragmatic difficulties in a subset of children. Development and Psychopathology, 12(2), 177–199. 10.1017/S0954579400002042 10847623

[bibr10-13591045231220694] Bishop D. V. M. Nation K. Patterson K. (2014). When words fail us : Insights into language processing from developmental and acquired disorders. Philosophical Transactions of the Royal Society of London - Series B: Biological Sciences, 369(1634), 20120403. 10.1098/rstb.2012.0403 24324244 PMC3866430

[bibr11-13591045231220694] Bishop D. V. M. Snowling M. J. Thompson P. A. Greenhalgh T. CATALISE-2 consortium (2017). Phase 2 of CATALISE : A multinational and multidisciplinary delphi consensus study of problems with language development : Terminology. Journal of Child Psychology and Psychiatry, 58(10), 1068–1080. 10.1111/jcpp.12721 28369935 PMC5638113

[bibr12-13591045231220694] Brezinka V. (2014). Computer games supporting cognitive behaviour therapy in children. Clinical Child Psychology and Psychiatry, 19(1), 100–110. 10.1177/1359104512468288 23258925

[bibr13-13591045231220694] Brinton B. Fujiki M. (2006). Social intervention for children with language impairment: Factors affecting efficacy. Communication Disorders Quarterly, 28(1), 39–41. 10.1177/15257401060280010501

[bibr14-13591045231220694] Carpendale J. I. M. Lewis C. (2004). Constructing an understanding of mind: The development of children’s social understanding within social interaction. Behavioral and Brain Sciences, 27(1), 79–151. 10.1017/S0140525X04000032 15481944

[bibr15-13591045231220694] Chiu A. W. Desai P. Skriner L. Catarozoli C. Sullivan P. Bennett S. M. (2022). Youth top problems in an acute psychiatric sample: Describing consumer-nominated treatment needs in an adolescent partial hospital setting. Child Psychiatry and Human Development. 10.1007/s10578-022-01427-3 36074210

[bibr16-13591045231220694] Choi J. Medalia A. (2005). Factors associated with a positive response to cognitive remediation in a community psychiatric sample. Psychiatric Services, 56(5), 602–604. 10.1176/appi.ps.56.5.602 15872171

[bibr17-13591045231220694] Conti-ramsden G. Mok P. L. H. Pickles A. Durkin K. (2013). Adolescents with a history of specific language impairment (SLI): Strengths and difficulties in social, emotional and behavioral functioning. Research in Developmental Disabilities, 34(11), 4161–4169. 10.1016/j.ridd.2013.08.043 24077068 PMC3830176

[bibr18-13591045231220694] Diamond A. (2013). Executive functions. Annual Review of Psychology, 64, 135–168. 10.1146/annurev-psych-113011-143750 PMC4084861 23020641

[bibr19-13591045231220694] Diamond A. Lee K. (2011). Interventions shown to aid executive function development in children 4 to 12 years old (Science (959)). Science, 334(6054), 311. 10.1126/science.334.6054.311-d PMC3159917 21852486

[bibr20-13591045231220694] Didehbani N. Allen T. Kandalaft M. Krawczyk D. Chapman S. (2016). Virtual reality social cognition training for children with high functioning autism. Computers in Human Behavior, 62, 703–711. 10.1016/j.chb.2016.04.033 PMC3536992 22570145

[bibr21-13591045231220694] Durkin K. Conti-ramsden G. (2010). Young people with specific language impairment: A review of social and emotional functioning in adolescence. Child Language Teaching and Therapy, 26(2), 105–121. 10.1177/0265659010368750

[bibr22-13591045231220694] Durkin K. Toseeb U. Botting N. Pickles A. Conti-Ramsden G. (2017). Social confidence in early adulthood among young people with and without a history of language impairment. Journal of Speech, Language, and Hearing Research: JSLHR, 60(6), 1635–1647. 10.1044/2017_JSLHR-L-16-0256 28586830 PMC5544415

[bibr23-13591045231220694] Durrleman S. (2020). Mentalizing: What’s language got to do with it? Language Acquisition, 27(3), 255–275. 10.1080/10489223.2020.1769624

[bibr25-13591045231220694] Fernyhough C. (2008). Getting Vygotskian about theory of mind: Mediation, dialogue, and the development of social understanding. Developmental Review, 28(2), 225–262. 10.1016/j.dr.2007.03.001

[bibr26-13591045231220694] Gerber S. Brice A. Capone N. Fujiki M. Timler G. (2012). Language use in social interactions of school-age children with language impairments: An evidence-based systematic review of treatment. Language, Speech, and Hearing Services in Schools, 43(2), 235–249. 10.1044/0161-1461(2011/10-0047 22052968

[bibr27-13591045231220694] Gorini A. Capideville C. S. De Leo G. Mantovani F. Riva G. Ph D. (2011). The role of immersion and narrative in mediated presence: The virtual hospital experience. Cyberpsychology, Behavior, and Social Networking, 14(3), 99–105. 10.1089/cyber.2010.0100 20649451

[bibr28-13591045231220694] Hart K. I. Fujiki M. Brinton B. Hart C. H. (2004). Social behavior and severity of Language impairment. Journal of Speech, Language, and Hearing Research, 47(3), 647–662. 10.1044/1092-4388(2004/050) 15212575

[bibr29-13591045231220694] Henry L. A. Messer D. J. Nash G. (2012). Executive functioning in children with specific language impairment. Journal of Child Psychology and Psychiatry, 53(1), 37–45. 10.1111/j.1469-7610.2011.02430.x 21668446

[bibr31-13591045231220694] Hughes D. M. Turkstra L. S. Wulfeck B. B. (2009). Parent and self-ratings of executive function in adolescents with specific language impairment. International Journal of Language and Communication Disorders, 44(6), 901–916. 10.3109/13682820802425693 19105067

[bibr32-13591045231220694] Kandalaft M. R. Didehbani N. Krawczyk D. C. Allen T. T. Chapman S. B. (2013). Virtual reality social cognition training for young adults with high-functioning autism. Journal of Autism and Developmental Disorders, 43(1), 34–44. 10.1007/s10803-012-1544-6 22570145 PMC3536992

[bibr33-13591045231220694] Kassai R. Futo J. Demetrovics Z. Takacs Z. K. (2019). A meta-analysis of the experimental evidence on the near- and far-transfer effects among children’s executive function skills. Psychological Bulletin, 145(2), 165–188. 10.1037/bul0000180 30652908

[bibr35-13591045231220694] Kuhn L. J. Willoughby M. T. Wilbourn M. P. Vernon-Feagans L. Blair C. B. Family Life Project Key Investigators (2014). Early communicative gestures prospectively predict language development and executive function in early childhood. Child Development, 85(5), 1898–1914. 10.1111/cdev.12249 24773289 PMC4165687

[bibr36-13591045231220694] Laugeson E. A. Frankel F. Gantman A. Dillon A. R. Mogil C. (2012). Evidence-based social skills training for adolescents with autism spectrum disorders: The UCLA PEERS program. Journal of Autism and Developmental Disorders, 42(6), 1025–1036. 10.1007/s10803-011-1339-1 21858588

[bibr37-13591045231220694] Law J. Boyle J. Harris F. Harkness A. Nye C. (2000). Prevalence and natural history of primary speech and language delay: Findings from a systematic review of the literature. International Journal of Language and Communication Disorders, 35(2), 165–188. 10.1080/136828200247133 10912250

[bibr38-13591045231220694] Law J. Dennis J. A. Charlton J. J. V. (2017). Speech and language therapy interventions for children with primary speech and/or language disorders. Cochrane Database of Systematic Reviews, 2017(3), 12490. 10.1002/14651858.CD012490 PMC8407295 12918003

[bibr39-13591045231220694] Mcquade J. D. Murray-close D. Shoulberg E. K. Hoza B. (2013). Working memory and social functioning in children. Journal of Experimental Child Psychology, 115(3), 422–435. 10.1016/j.jecp.2013.03.002 23665178

[bibr40-13591045231220694] Milligan K. Astington J. W. Dack L. A. (2007). Language and theory of mind meta-analysis of the relation between Language ability and false-belief understanding. Child Development, 78(2), 622–646. 10.1111/j.1467-8624.2007.01018.x 17381794

[bibr41-13591045231220694] Miyake A. Friedman N. P. Emerson M. J. Witzki A. H. Howerter A. Wager T. D. (2000). The unity and diversity of executive functions and their contributions to complex “frontal lobe” tasks: A latent variable analysis. Cognitive Psychology, 41(1), 49–100. 10.1006/cogp.1999.0734 10945922

[bibr42-13591045231220694] Mok P. L. H. Pickles A. Durkin K. Conti-ramsden G. (2014). Longitudinal trajectories of peer relations in children with specific language impairment. Journal of Child Psychology and Psychiatry, 55(5), 516–527. 10.1111/jcpp.12190 24410167 PMC4283728

[bibr43-13591045231220694] Nijman S. A. Veling W. Greaves-lord K. Vermeer R. R. Vos M. Zandee C. E. R. Zandstra D. C. Geraets C. N. W. Pijnenborg G. H. M. (2019). Dynamic interactive social cognition training in virtual reality (DiSCoVR) for social cognition and social functioning in people with a psychotic disorder: Study protocol for a multicenter randomized controlled trial. BioMed Central psychiatry, 19(1), 272. 10.1186/s12888-019-2250-0 31488103 PMC6727396

[bibr44-13591045231220694] Nijman S. A. Veling W. Greaves-lord K. Vos M. Zandee C. E. R. Aan Het Rot M. Geraets C. N. W. Pijnenborg G. H. M. (2020). Dynamic interactive social cognition training in virtual reality (DiSCoVR) for people with a psychotic disorder: Single-group feasibility and acceptability study. Schizophrenia Bulletin, 7(8), Article e17808. 10.2196/17808 PMC7442939 32763880

[bibr45-13591045231220694] Nilsson K. K. de López K. J. (2016). Theory of mind in children with specific language impairment: A systematic review and meta-analysis. Child Development, 87(1), 143–153. 10.1111/cdev.12462 26582261

[bibr46-13591045231220694] Orsmond G. I. Cohn E. S. (2015). The distinctive features of a feasibility study: Objectives and guiding questions. OTJR: Occupation, Participation and Health, 35(3), 169–177. 10.1177/1539449215578649 26594739

[bibr47-13591045231220694] Parsons S. Mitchell P. (2002). The potential of virtual reality in social skills training for people with autistic spectrum disorders. Journal of Intellectual Disability Research, 46(Pt 5), 430–443. 10.1046/j.1365-2788.2002.00425.x 12031025

[bibr65-9135910452313] Saarni C. (1999). The development of emotional competence. New York: Guilford Press.

[bibr70-9135910452318] Schlichting L. Lutje Spelberg (2010a). Schlichting test voor taalbegrip [Schlichting Test for Language Comprehension]. Bohn Stafleu van Loghum.

[bibr71-9135910452319] Schlichting L. Lutje Spelberg (2010b). Schlichting test voor taalproductie [Schlichting Test for Language Production] (2nd ed.). Bohn Stafleu van Loghum.

[bibr69-9135910452317] SIAC (2023). Kwaliteitskader SIAC: Voor zorg geleverd vanuit de Zvw aan cliënten met een auditieve en/of communicatieve beperking [Quality framework SIAC: For care provided by the healthcare insurance act to clients with an auditory and/or communicative disability]. http://www.siac.nu/site/assets/files/1034/kwaliteitskader_siac_2022_versie_16_februari_2023.pdf.

[bibr48-13591045231220694] Sikora K. Roelofs A. Hermans D. Knoors H. (2019). Executive control in language production by children with and without language impairment. International Journal of Language & Communication Disorders, 54(4), 645–655. 10.1111/1460-6984.12470 30920093

[bibr49-13591045231220694] Smit L. Knoors H. Hermans D. Verhoeven L. Vissers C. (2019). The interplay between theory of mind and social emotional functioning in adolescents with communication and language problems. Frontiers in Psychology, 10(JULY), 1488. 10.3389/fpsyg.2019.01488 31333537 PMC6616194

[bibr67-9135910452315] St Clair M. C. Pickles A. Durkin K. Conti-Ramsden G. (2011). A longitudinal study of behavioral, emotional and social difficulties in individuals with a history of specific language impairment (SLI). Journal of Communication Disorders, 44. 10.1016/j.jcomdis.2010.09.004 20970811

[bibr51-13591045231220694] Timler G. R. Olswang L. B. Coggins T. E. (2005). Do I know what I need to do?” A social communication intervention for children with complex clinical profiles. Language, Speech, and Hearing Services in Schools, 36(1), 73–85. 10.1044/0161-1461(2005/007) 15801509

[bibr52-13591045231220694] Timler G. R. Vogler-Elias D. McGill K. F. (2007). Strategies for promoting generalization of social communication skills in preschoolers and school-aged children. Topics in Language Disorders, 27(2), 167–181. 10.1097/01.TLD.0000269931.18881.90

[bibr53-13591045231220694] Tomas E. Vissers C. (2019). Behind the scenes of developmental language disorder: Time to call neuropsychology back on stage. Frontiers in Human Neuroscience, 12(January), 1–10. 10.3389/fnhum.2018.00517 PMC6333853 30687040

[bibr54-13591045231220694] Van Agt H. Verhoeven L. Van Den Brink G. De Koning H. (2011). The impact on socio-emotional development and quality of life of language impairment in 8-year-old children. Developmental Medicine and Child Neurology, 53(1), 81–88. 10.1111/j.1469-8749.2010.03794.x 20875040

[bibr72-9135910452321] van Hoof A. Luteijn E. Quist Y. Wagenaar I. de Wit J. (2023). Ik en de ander vragenlijst [IKAN]. Kentalis.

[bibr56-13591045231220694] Veling W. Moritz S. van der Gaag M. (2014). Brave new worlds — review and update on virtual reality assessment and treatment in psychosis. Schizophrenia Bulletin, 40(6), 1194–1197. 10.1093/schbul/sbu125 25193975 PMC4193729

[bibr57-13591045231220694] Verhoef R. E. J. van Dijk A. Verhulp E. E. de Castro B. O. (2021). Interactive virtual reality assessment of aggressive social information processing in boys with behaviour problems: A pilot study. Clinical Psychology and Psychotherapy, 28(3), 489–499. 10.1002/cpp.2620 34048619 PMC8361679

[bibr66-9135910452314] Vissers C. Isarin J. Hermans D. Jekeli I. (2021). Taal in het kwadraat: kinderen met TOS beter begrijpen. Huizen: Pica.

[bibr58-13591045231220694] Vissers C. Koolen S. (2016). Theory of mind deficits and social emotional functioning in preschoolers with specific language impairment. Frontiers in Psychology, 7(NOV), 1–7. 10.3389/fpsyg.2016.01734 27867370 PMC5095688

[bibr59-13591045231220694] Vissers C. Koolen S. Hermans D. Scheper A. Knoors H. (2015). Executive functioning in preschoolers with specific language impairment. Frontiers in Psychology, 6(OCT), 1–8. 10.3389/fpsyg.2015.01574 26539136 PMC4611093

[bibr61-13591045231220694] Vugs B. Knoors H. Cuperus J. Hendriks M. Verhoeven L. (2015). Interactions between working memory and language in young children with specific language impairment (SLI). Child Neuropsychology, 22(8), 955–978. 10.1080/09297049.2015.1058348 26144244

[bibr62-13591045231220694] Westby C. Robinson L. (2014). A developmental perspective for promoting theory of mind. Topics in Language Disorders, 34(4), 362–382. 10.1097/TLD.0000000000000035

[bibr63-13591045231220694] Wiefferink K. van Beugen C. Wegener Sleeswijk B. Gerrits E. (2020). Children with language delay referred to Dutch speech and hearing centres : Caseload characteristics. International Journal of Language and Communication Disorders, 55(4), 573–582. 10.1111/1460-6984.12540 32459389 PMC7383695

[bibr64-13591045231220694] Wiig E. Semel E. Secord W. E. (2019). CELF-5-NL: Clinical evaluation of Language Fundamentals – fifth edition – nederlandstalige versie. Pearson.

