# Supplementary material for: Interactive virtual reality training to improve socio-emotional functioning in adolescents with developmental language disorders: A feasibility study
Source: Clin Child Psychol Psychiatry. 2023 Dec 21;29(3):1100–20. doi: 10.1177/13591045231220694 (PMC11188569; doi:10.1177/13591045231220694)
Supplement: Supplemental Material - Interactive virtual reality training to improve socio-emotional functioning in adolescents with developmental language disorders: A feasibility study [file sj-pdf-1-ccp-10.1177_13591045231220694.pdf]

Conversation

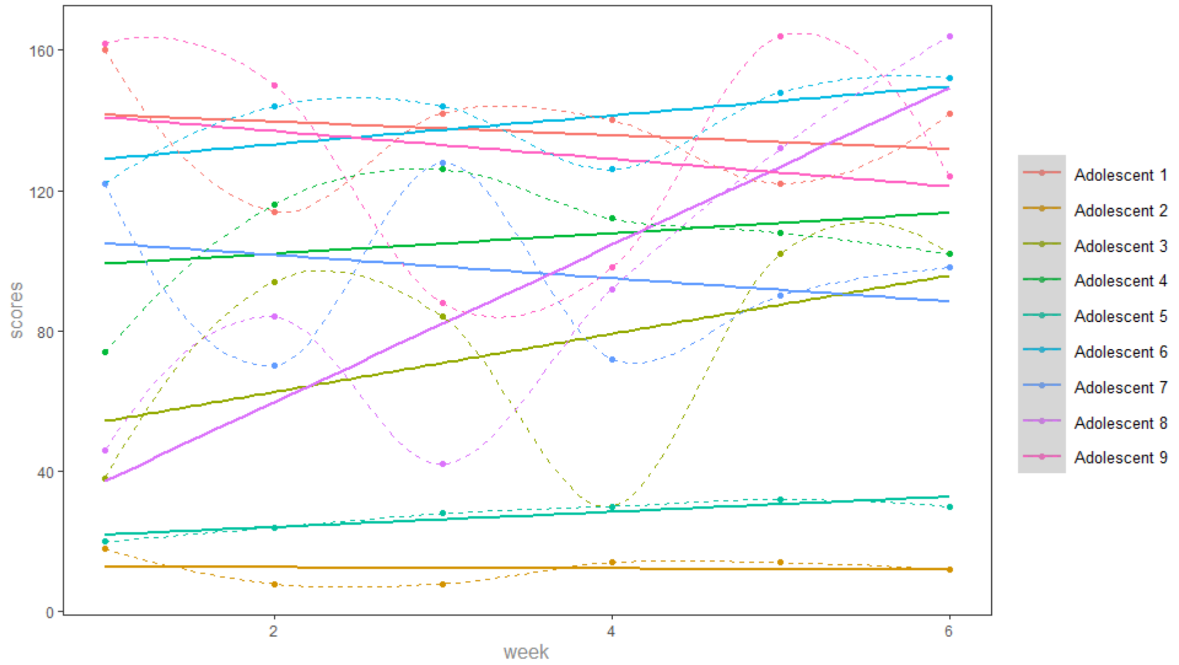

Weekly reported socio-emotional functioning per skill. Dots reflect reported scores and solid lines reflect linear trendlines.

Question

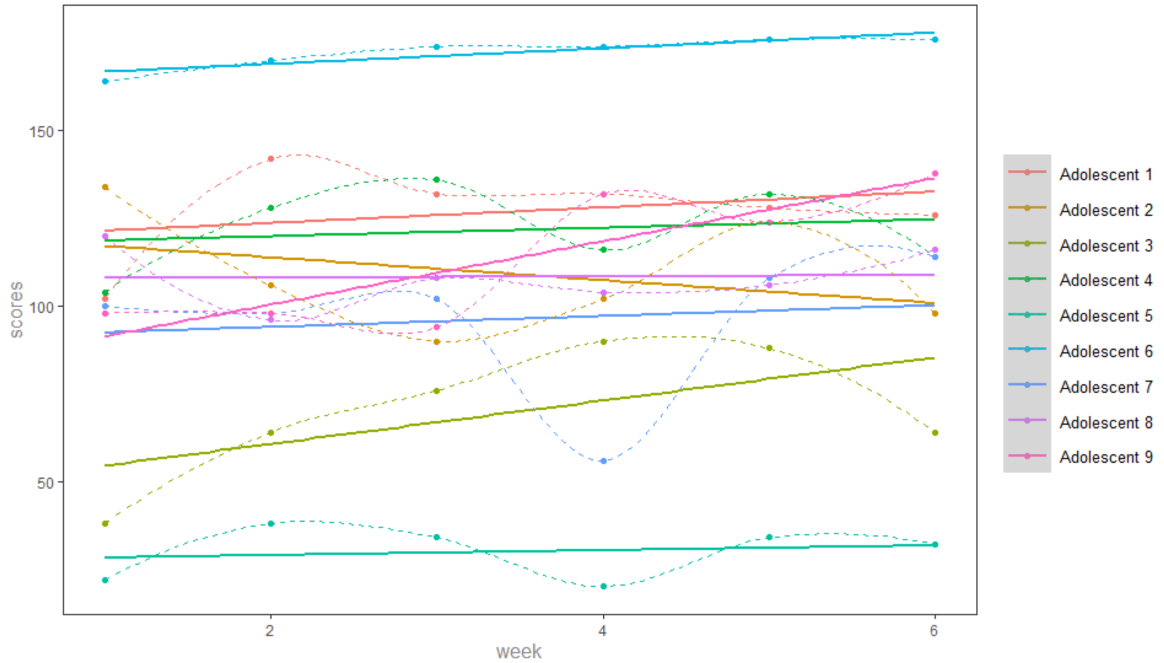

Weekly reported socio-emotional functioning per skill. Dots reflect reported scores and solid lines reflect linear trendlines.

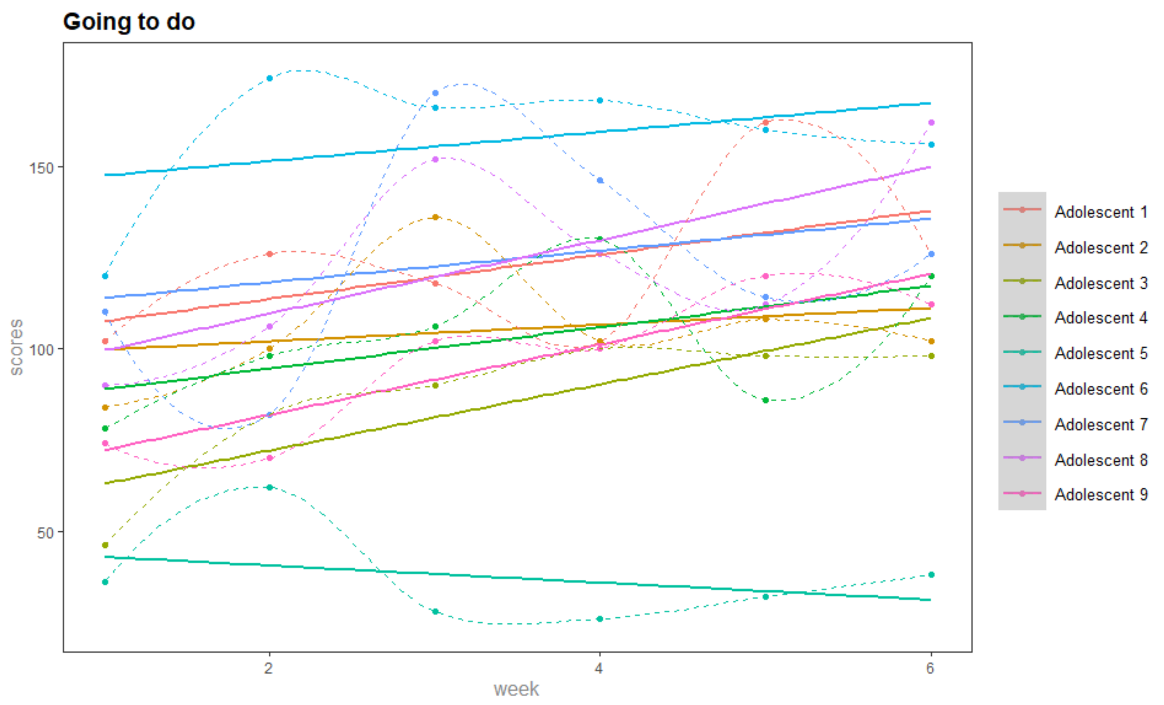

Weekly reported socio-emotional functioning per skill. Dots reflect reported scores and solid lines reflect linear trendlines.

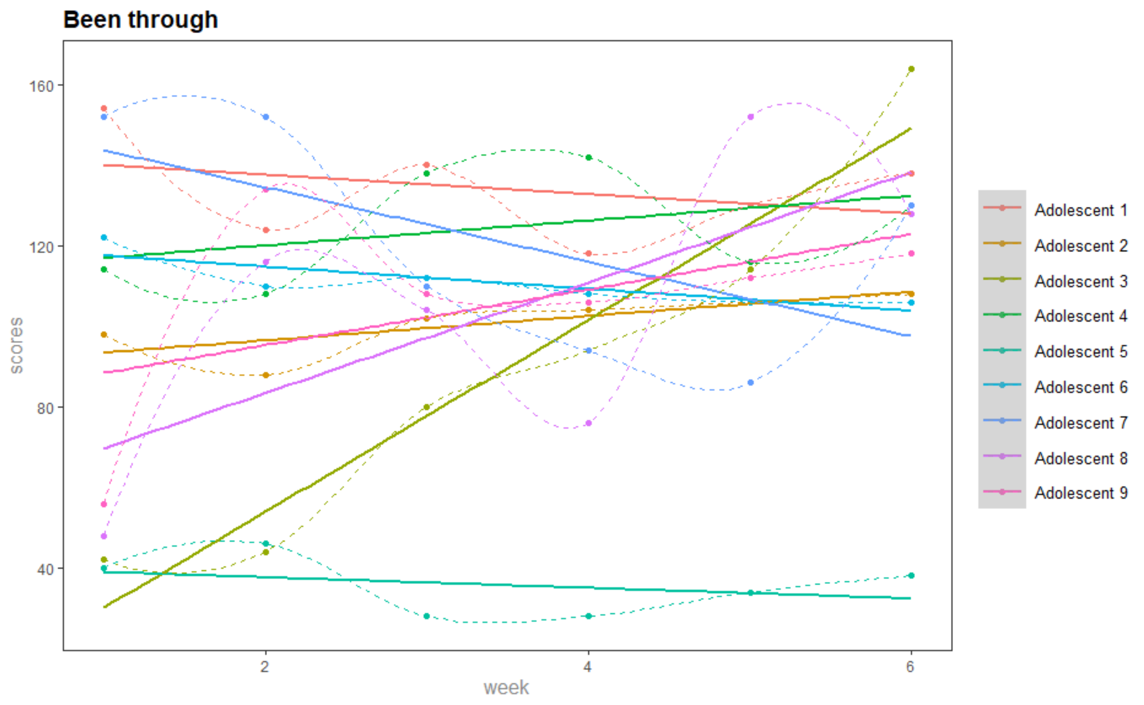

Weekly reported socio-emotional functioning per skill. Dots reflect reported scores and solid lines reflect linear trendlines.

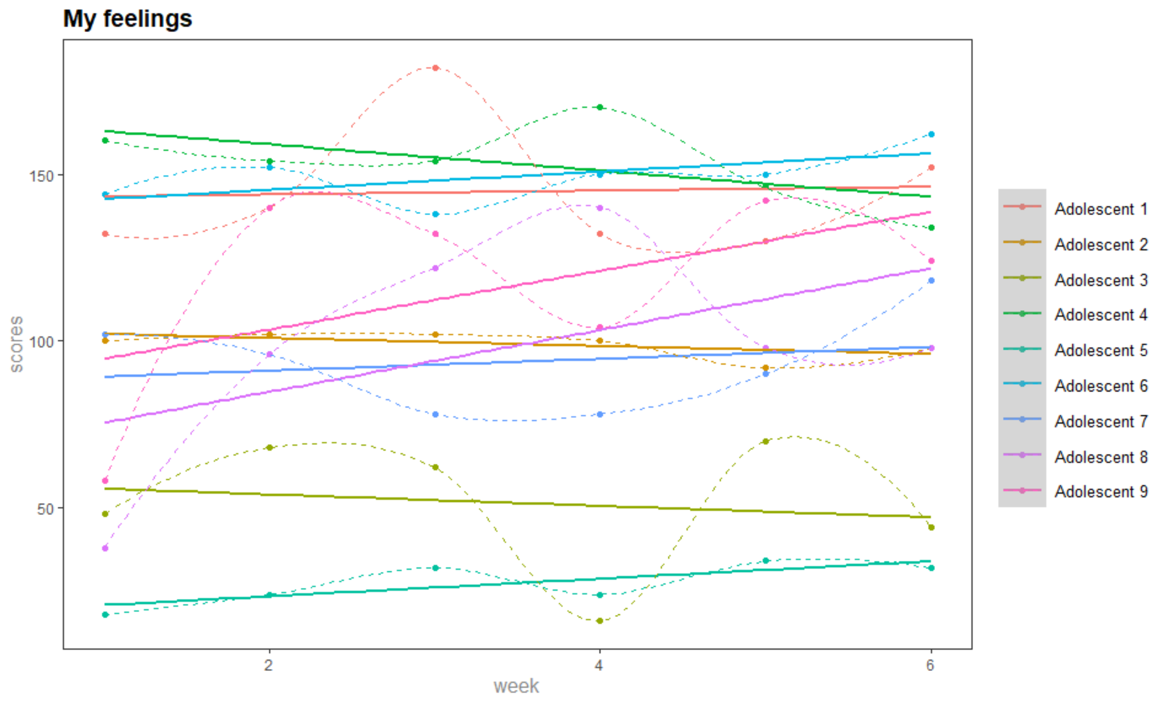

Weekly reported socio-emotional functioning per skill. Dots reflect reported scores and solid lines reflect linear trendlines.

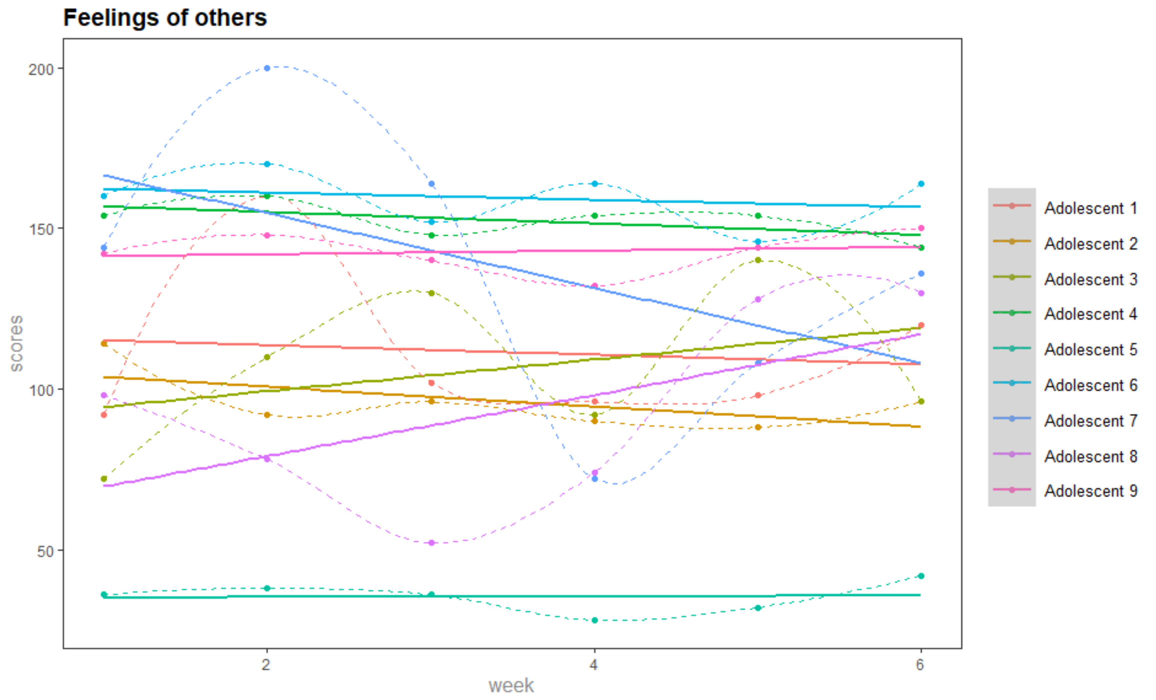

Weekly reported socio-emotional functioning per skill. Dots reflect reported scores and solid lines reflect linear trendlines.

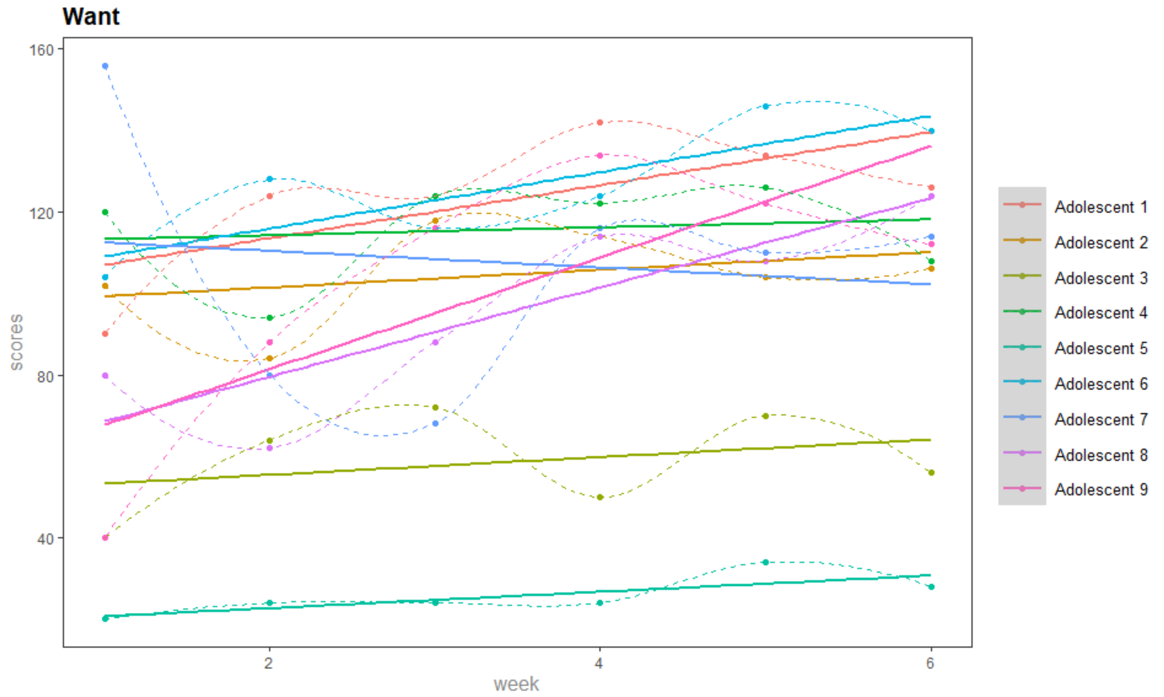

Weekly reported socio-emotional functioning per skill. Dots reflect reported scores and solid lines reflect linear trendlines.

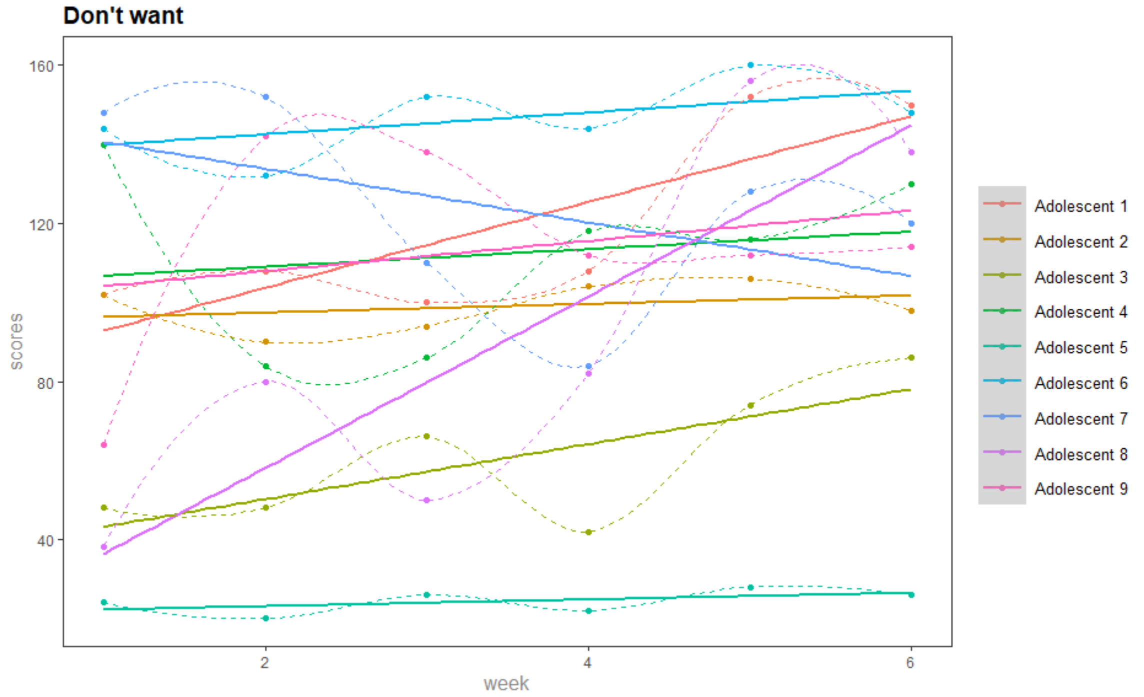

Weekly reported socio-emotional functioning per skill. Dots reflect reported scores and solid lines reflect linear trendlines.
